# Systematic review of the impact of ginger extract and alpinetin on pregnancy outcomes in animal models

**DOI:** 10.1186/s12906-025-04904-z

**Published:** 2025-05-16

**Authors:** Jonquile T. Williams, Kendra A. Tiani, Margaret J. Foster, Amanda J. MacFarlane, Regan L. Bailey, Patrick J. Stover, Martha S. Field

**Affiliations:** 1 Division of Nutritional Sciences, Cornell University, Ithaca, NY USA; 2 Institute for Connecting Nutrition and Health, Florida State University, Tallahassee, FL USA; 3 Center for Systematic Reviews and Evidence Syntheses, Texas A&M University, College Station, TX USA; 4 Nutrition Research Division, Health Canada, Ottawa, ON Canada; 5 Department of Behavioral Science and Social Medicine, Florida State University, Tallahassee, FL USA; 6 Department of Biomedical Sciences, Florida State University, Tallahassee, FL USA

**Keywords:** Ginger, Ginger extract, Pregnancy, Animal, Lactation

## Abstract

**Background:**

The objective of this systematic review was to evaluate existing scientific evidence regarding the effectiveness and safety of preparations of bioactive compounds of the Zingiberaceae family in animal models during gestation and lactation.

**Methods:**

A systematic protocol was registered with the Open Science Framework (OSF) (10.17605/OSF.IO/ADU68). The literature search was conducted on selected databases such as MEDLINE, Embase, Center for Agricultural and Biosciences International, and the International Pharmaceutical Abstracts databases. The full search strategy is included in the Supplementary Materials. Main keywords related to population included terms related to pregnancy and lactation; keywords related to intervention included key terms for alpinetin, ginger, and *Zingiberaceae* plants. We included maternal (i.e., dam) and neonatal (i.e., pup) outcome(s) reported in studies with ginger preparations in various forms given during pregnancy or lactation compared to placebo. Risk of bias was assessed using the Systematic Review Center for Laboratory animal experimentation (SYRCLE) risk of bias tool.

**Results:**

Twelve studies published between 2000 and 2022 were included in the review. Ginger and its bioactive compounds, [6]-gingerol, [8]-gingerol, [10]-gingerol, and [6]-shogaol, were found to have protective effects against gestational and developmental toxicities. This included mitigating and preventing organ toxicity (e.g., liver and kidney), improved gestational weight gain, and improved placental function; fetal benefits included prevention of organ damage (e.g., liver, kidney, cardiac), improved fetal growth, reduced oxidative stress, and reduced death. In studies involving toxic exposures such as heavy metals and pesticides, ginger mitigated adverse effects on maternal and fetal health, improving outcomes such as placental function birth weight, and organ development (e.g., liver, kidney, cardiac). Alpinetin, a flavonoid rich in ginger plants, showed anti-inflammatory effects in lactation by reducing cytokine levels and improving mammary tissue health. Studies on fetal development reported improvements in birth weight, growth metrics, and reductions in death rates when ginger was administered at moderate doses, specifically ginger tea 20 g/L-50 g/L or gingerol 25 mg/kg/body weight. However, higher doses (specifically, 50 mg alligator pepper, 2,000 mg/kg body weight *Zingiber officinale*) caused adverse reproductive outcomes such as reduced weight gain (< 50%), maternal toxicity, disrupted estrous cycle, and increased fetal death. Sensitivity analysis confirmed that lower dosages of rhizome-derived ginger preparations (*Zingiber officinale*) (< 200 mg/kg/day) were safer.

**Conclusion:**

The majority of the included studies reported protective effects of lower dose *Zingiberaceae* preparations (< 200 mg/kg/day) on gestational and developmental toxicities in animal models. Standardization of ginger interventions and more robust study designs are needed to optimize ginger form, amounts, preparation, doses, and timing of exposures to understand how maximize benefits while minimizing potential adverse effects in animal models before such data can be translated meaningfully to humans.

**Clinical trial number:**

Not applicable.

**Supplementary Information:**

The online version contains supplementary material available at 10.1186/s12906-025-04904-z.

## Introduction

Plants of the *Zingiberaceae* family are widely used spices and medicinal plants. Ginger (*Zingiber officinale*) also belongs to the *Zingiberaceae* family and is known for its distinct flavor and numerous potential health benefits including anti-inflammatory, antiemetic, and digestive properties [1]. Ginger is frequently consumed as a common remedy for nausea and digestive disturbances [1, 2] including during pregnancy, with the most common use of 1000 mg/day of ginger capsules [3]. The phenolic compounds, including gingerols, shogoals, and paradols [1] and alpinetin, are believed to contribute to the medicinal properties of ginger and ginger extract [4].

Alpinetin is a flavonoid compound predominantly found in the seeds from plants of the Alpinia genus, which is also from the *Zingiberaceae* family, and it is also a constituent of ginger that has gained attention for its potential anti-inflammatory, antioxidant [5], and anti-cancer properties [6]. In the context of pregnancy, the anti-inflammatory properties of alpinetin may be beneficial in reducing inflammation-related complications at a dosage of 10–50 mg alpinetin per kg body weight [6].

A recent umbrella review of 22 meta-analyses of intervention studies including 22 independent human studies found that ginger preparation interventions during pregnancy and lactation had a significant beneficial effect on symptoms of nausea but not vomiting. The authors noted that most studies reported insufficient detail for ginger formulations, dosages, and intervention duration limiting the generalizability of study results [3].

Despite the promising findings from human trials, significant gaps in knowledge remain about safety and efficacy during the perinatal period [6]. Although antioxidant and anti-inflammatory effects have been suggested, detailed mechanistic studies are lacking [6]. There is a need to investigate treatment dose and duration to maximize benefits while minimizing potential adverse effects. Experimental animal model systems allow for more rapid data collection and a more comprehensive understanding of complex molecular interactions among maternal dietary components and maternal/offspring health outcomes and allow for a safety assessment of ginger preparations to inform future human intervention studies. Simply stated, interventions in animal models are more amenable to research questions for perinatal human health for highlighting complex molecular mechanisms and for ethical considerations.

The primary objectives of this systematic review were to: (1) determine the extent and quality of the existing evidence regarding the effectiveness and safety of *Zingiberaceae* preparations during gestation and lactation in animal models; (2) identify the range of maternal and fetal outcomes of *Zingiberaceae* preparations assessed in animal models during pregnancy and lactation; and (3) determine if factors such as timing of supplementation, duration of treatment, dosage, form, and animal model health status influence the efficacy and safety of *Zingiberaceae* preparations exposure during pregnancy and lactation. By summarizing the current state of the evidence of mechanistic studies examining the effect of ginger preparations on maternal and offspring health outcomes in animal models, this review will identify evidence gaps to inform future research directions and potential clinical studies.

## Methods

### Protocol and registration

This review followed the Preferred Reporting Items for Systematic Reviews and Meta-Analyses (PRISMA) protocol for systematic reviews [7]. The PRISMA checklist is available in the supplemental information. The systematic review protocol was registered in the Open Science Framework (OSF) (10.17605/OSF.IO/ADU68).

### Search strategy

A search strategy was employed to capture the full scope of the literature to identify primary animal model studies that examined the effectiveness and safety of *Zingiberaceae* preparations during gestation and lactation (Supplementary Material).Databases searched included MEDLINE, Embase, Center for Agricultural and Biosciences International Abstracts, and the International Pharmaceutical Abstracts databases. See the full search strategy in supplemental information. Reference lists of included studies were manually searched for additional eligible studies not retrieved by our search. The last search date was April 10, 2023, for all of the databases, consistent with the timeline listed in the registration.

### Study selection criteria

Covidence systematic review software (Veritas Health Innovation, Melbourne, Australia) was used to manage the systematic literature search results, including importing and de-duplicating articles. This software facilitated the importation of search findings and the identification and removal of duplicate articles. Articles were screened by title and abstract and then by full text (MF, KT, JW, TU, JA, RS, JU, PH). Two reviewers independently screened the studies in parallel. A third reviewer was consulted to resolve disagreements.

The Population, Intervention, Comparison, and Outcome (PICO) framework was used to establish the inclusion and exclusion criteria for this review. All full-text peer-reviewed articles in the English language up to December 2023 were eligible for inclusion. (1) Population: included “models” were defined as a non-human mammalian species, including rats, mice, swine, primates, and rabbits, of reproductive age that were pregnant or lactating. Included studies encompassed both “healthy” animals and those with induced conditions relevant to maternal, fetal, and offspring outcomes (e.g., hypertension, fetal developmental impairment, gestational weight gain, toxicity, and teratogenicity). (2) “Interventions” included used various forms of preparations from plants of the Zingirberacae family to examine the effect on the model organism’s health outcomes; (3) Control or comparator: control involves untreated animal models or those receiving standard care to assess the efficacy of the primary treatment. Included studies were required to implement *Zingiberaceae* preparations (ginger, ginger extract or other ginger bioactive component alpinetin) as an intervention and offer a valid comparison to a control group, such as untreated animals or those receiving a placebo. (4) Included outcomes assessed: maternal, fetal, or offspring function during pregnancy, lactation, or postnatal periods, e.g., gestational weight gain, fetal development, toxicity, or biomarkers thereof. Exclusion criteria for study selection included animal species outside the chosen scope, such as ruminants, amphibians, reptiles, fish, chickens, non-pregnant and non-lactating animals, or those concentrating solely on offspring receiving supplementation. In addition, non-animal models, including ex vivo, in vitro, and in silico studies were excluded; Studies without control groups, or those with non-comparable interventions (i.e. studies which would not allow for isolation of the effect of Zingiberaceae preparations), were excluded;

Additionally, this review included articles in English only. To ensure comprehensive coverage, the reference lists of included studies were searched for additional eligible studies not identified by our search.

### Data extraction

Two review authors independently (MF, KT) extracted data from the 12 included studies using a standardized, pre-piloted extraction template. Disagreements were resolved by consultation with a third reviewer (JW).

For each study we extracted comprehensive data including study identification details (author(s), year of publication, journal), study characteristics (study design, sample size, duration of study, funding sources), population details (species, strain, sex, age, health status, number of animals per group, pregnancy or lactation status), intervention details (type of supplementation, dosage, frequency, timing of intervention during pregnancy, method of administration, duration of intervention), control details (description of control group(s), type of control intervention), outcome measures (relevant maternal, fetal, and offspring health outcomes), results (quantitative data on outcomes, statistical analyses, effect sizes, confidence intervals, p-values), and risk of bias indicators (randomization, blinding, attrition rates, any reported biases).

All relevant details were extracted and recorded regarding maternal, fetal, or offspring health measures and outcomes. Maternal outcomes included body weight (weight gain/weight loss), organ weight (liver, placenta, kidney), markers of vascular function, immune response indicators (e.g., metabolites, cytokines), nutritional status and metabolic markers, length of gestation (in days) or litter size, lactation outcomes (e.g., milk volume), and indicators of toxicity or protection against toxicity. Fetal characteristics included birth weight, body weight, growth trajectory, fetal survival rates, organ development and weight, markers of fetal development (e.g., brain and skeletal), and indicators of toxicity or protection against toxicity. Offspring categories included postnatal growth trajectories (e.g., weight gain), immune function (e.g., metabolites, inflammation), and long-term health outcomes (e.g., development of metabolic or cardiovascular diseases). Additionally, reviewers described “other” health outcomes and information.

### Risk of bias assessment

Risk of bias (ROB) assessment was conducted independently by two reviewers (MF, JW) using the Systematic Review Center for Laboratory Animal Experimentation (SYRCLE) ROB tool for animal interventional studies [8]. This predefined checklist was used to assess selection bias, performance bias, detection bias, attrition bias, reporting bias and other bias.

### Data synthesis

While the original goal was to conduct quantitative synthesis through meta-analysis. data were synthesized narratively given the heterogeneity of the studies included. The narrative synthesis was conducted following the Synthesis Without Meta-analysis (SWiM) reporting guidelines [9] with a focus on describing the direction and magnitude of effects and detailing any observed patterns or discrepancies among the studies.

## Results

**Fig. 1:**
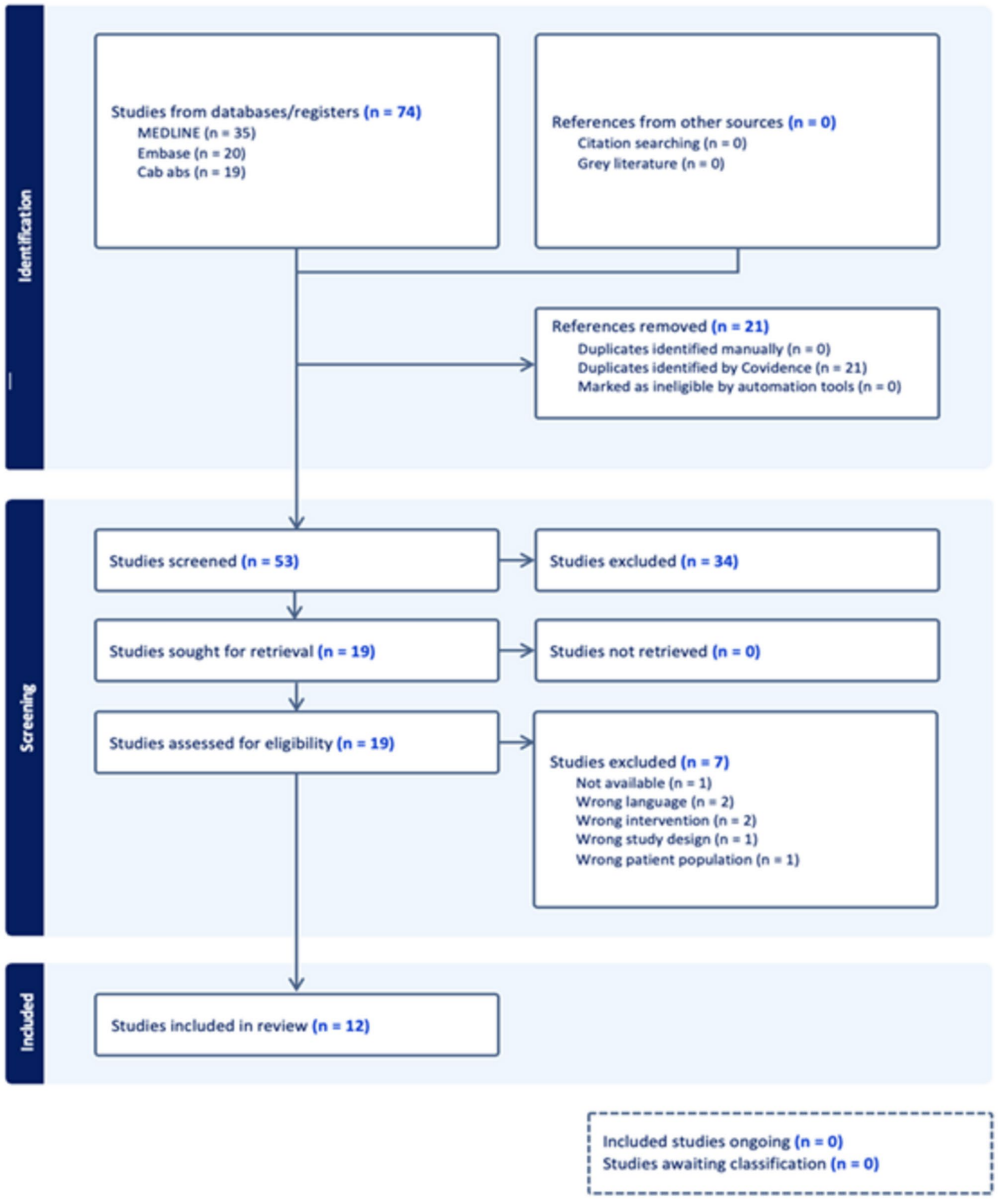
PRISMA 2020 flow diagram. Flow diagram of the identification and screening of studies included in the systematic review. Figure created using Covidence software

The literature search identified 74 studies (Fig. 1). Twenty-one duplicate articles were removed. Fifty-three studies were screened at the title and abstract stage, with 19 articles undergoing full text screening. During the full-text assessment, 7 studies were excluded due to non-availability (1 study), lack of availability in the English language (2 studies), irrelevant interventions (2 studies), unsuitable study design (1 study), and wrong patient population e.g., the study did not include an animal model (1 study)(Supplementary Table 1). A total 12 studies were included in the final review (Table 1).

**Table 1:** Summary of articles selected to evaluate the effectiveness of Ginger, ginger extract, or Alpenitin during pregnancy and lactation

Study	Model	Treated/Control	Intervention(s)	Supplementation Period	Maternal Outcomes	Neonatal Outcomes	Summary of Findings
**Studies with multiple outcomes**							
Balubaid, 2010	Rat (Rattus norvegicus)	3 / 1	1. control 2. 0.07 mg tetracycline/kg/day 3. therapeutic dose of ginger 4. 0.07 mg tetracycline/kg/day + similar dose of ginger ale *Treated daily one week prior to pregnancy. Route of administration not clear.	One week before pregnancy	Body weight Liver weight	Embryo number Embryo weight Embryo liver weight Congenital anomaly	The authors concluded that tetracycline decreased dam and embryo weight (and liver weight of each) and that ginger ale rescued the effects of tetracycline on these outcomes. They also reported no congenital anomalies in offspring. The presentation of the data and lack of statistical rigor make interpreting these conclusions difficult.
El Mazoudy, 2018	Mouse (ICR-CD1)	5 / 1	Dose response of ginger (*Zingiber officinale roscoe*, aqueous extract) ranging from 250–2000 mg/kg	Not specified	Fertility Weight status Toxicity	Fetal death Developmental outcomes Toxicity	The highest two doses of ginger (1000 and 2000 mg/kg/day) caused decreased maternal weight gain (*p* ≤ 0.05) and decreased food consumption (not clear that this is gestational weight or not). Estrous cycle length increased (*p* ≤ 0.05) and total estrous cycles decreased with the highest dose of ginger (2000 mg/kg/day); diestrus index also decreased in this group (*p* ≤ 0.05).The highest ginger dose resulted in decreased implantation and pre- and post-implantation fetus loss (*p* ≤ 0.05).Crown-rump length also decreased in the highest ginger exposure group (*p* ≤ 0.05).
Haijin, 2013	Mouse (Balb/c)	3 / 1	Lipopolysaccharide (LPS)-induced mastitis model + dose response (10, 25, 50 mg/kg IP alpinetin; with alpinetin given 1 h before LPS stimulation)	1 h before LPS stimulation	Lactation Immune function		Alpinetin decreased LPS-induced myeloperoxidase (MPO) activity in a dose-response fashion and reduced inflammatory cytokine levels (TNFa, IL-1B, IL-6) after LPS treatment in mammary tissue homogenate. Statistical representations are shown but did not address dose-response specifically.
Weidner, 2001	Rat (Wister SPR)	4 / 1	1. Control 2. 100 mg/kg/d *Zingiber officinale* ethanol extract EV.EXT 33 (eurovita extract) 3. 300 mg/kg/d EV.EXT 33 4. 1000 mg/kg/d EV.EXT 33 *Delivered via gastric intubation on days 6–15 of gestation	Gestation (days 6–15)	Fertility Gestational weight gain Mortality Toxicity	Developmental outcomes	Maternal body weight was lower in the 1000 mg/kg/d dose at 22 days gestation (*p* < 0.05). No effect of EV.EXT 33 on implantations, fertility index, number of corpora lutea, implantation loss, resorptions, number of fetuses born, uterine weight, or placental weight. No effect of EV.EXT 33 on skeletal malformations.
Wilkinson, 2000	Rat (Sprague Dawley)	2 / 1	1. control 2. 20 g/L ginger tea in drinking water 3. 50 g/L ginger tea in drinking water *Administered days 6–15 of gestation	Gestation (days 6–15)	Fertility Gestational weight gain Toxicity	Birth weight Fetal death Developmental outcomes	Ginger tea decreased fluid consumption at highest (50 g/L) dose (*p* < 0.01), not clear how this may affect results. Ginger tea did not affect gestational weight gain; did not affect maternal liver, kidney, placenta, or uterus weight. Ginger tea did not affect implantations/dam but did increase resorptions (*p* < 0.05). Fetal weight was increased by ginger tea (*p* < 0.01); crown-rump length was increased in males only in the 50 g/L exposure group (*p* < 0.01).
Studies with primarily toxicity prevention outcomes							
EL-Aziz, 2018	Rat (Sprague-Dawley)	4 / 1	1. Control 2. *Zingiber officinale* 3. CdCl_2_ 4. *Zingiber officinale* + CdCl_2_ *Given through oral intragastric tube during either gestation or gestation and lactation (depending on study group).	During gestation or gestation + lactation	Weight gain Toxicity prevention	Toxicity prevention	Zingiber officinale rescued gestational weight gain, which was severely reduced by CdCl_2_ exposure (*p* < 0.001); partially rescued effects of ClCl_2_ on gravid uterine weight and placental weight (*p* < 0.001). Histology images are shown for maternal and fetal liver and kidney, but findings from images are not quantified. However, authors state that CdCl_2_-induced damage to these tissues was improved/prevented by zingiber officinale.
El-Borm, 2021a	Rat (Wistar)	3 / 1	1. control 2. ginger (water extract of ginger, 200 mg/kg) 3. labetalol (medication used to treat hypertension during pregnancy, 300 mg/kg) 4. ginger + labetalol *Given through oral injection during organogenesis (6th -15th day of gestation)	Organogenesis (days 6–15 of gestation)		Toxicity prevention	Labetalol caused changes in offspring cardiac muscle physiology, as evidenced by histology, and authors state that this was rescued by ginger (findings not quantified); these findings were confirmed by electron microscopy (also not quantified). Flow cytometry revealed more apoptotic (*p* < 0.001), and fewer viable, fetal cardiac cells as a result of labetalol, and these outcomes were partially rescued by ginger.
El-Borm, 2021b	Rat (Wistar)	3 / 1	1. control 2. ginger (water extract of ginger, 200 mg/kg) 3. labetalol (medication used to treat hypertension during pregnancy, 300 mg/kg) 4. ginger + labetalol *Given through oral injection (6th -20th day of gestation)	Gestation (days 6–20)	Toxicity prevention		In maternal cardiac tissue labetalol induced histological changes including disorganization of cardiac fibers and blood vessel dilation and congestion; these outcomes were rescued by ginger (findings not quantified); similar observations were seen using electron microscopy (findings not quantified). Labetalol increased apoptosis in maternal cardiac muscle as measured by caspase-3 immunohistochemistry (*p* < 0.001), and the caspase-3 staining was partially rescued in the labetalol + ginger group (*p* < 0.001). Labetalol decreased placental weight, and this was rescued in the labetalol + ginger group. Similar histological, electron microscopy, and immunohistochemical (caspase-3) findings were observed in the placenta as in maternal cardiac muscle. Only caspase-3 staining was quantified, though again it was elevated by labetalol and rescued in the labetalol + ginger group.
Farag, 2010	Rats (albino)	8 / 1	1. control 2. 1% (w/2) ginger 3. lead acetate (120 ug/animal/d) 4. fenitrothion (pesticide; 10 mg/kg/d) 5. lead + fenitrothion 6. fenitrothion + ginger 7. lead + ginger 8. lead + fenitrothion + ginger	During pregnancy	Gestational weight gain Toxicity prevention	Birth weight Fetal death Developmental outcomes Toxicity prevention	All combination treatment groups (including those with ginger) increased gestational weight gain (*p* < 0.05). Ginger alone affected some plasma/blood biomarkers of liver function, but not to the extent that the combination treatments affected these parameters. Uterine morphology largely unchanged: number of corpora lutea per dam was increased in combination groups (*p* < 0.05) but not by ginger alone. Mean fetal weight and placental weight (*p* < 0.05) were also increased in combination groups, but not by ginger alone. Skeletal malformations are noted in the combination groups but are not characterized statistically; it does not look like ginger is causing malformations or that it is rescuing malformations caused by the other two agents.
Othman, 2022	Mouse	3 / 1	1. control 2. 350 mg/kg/day body weight methyl ethyl ketone (MEK) 3. 350 mg/kg/day MEK + 25 mg/kg/day gingerol	During gestation	Gestational weight gain Toxicity prevention	Birth weight Developmental outcomes Toxicity prevention	Gestational weight gain was reduced by MEK exposure (*p* < 0.01), and this was rescued by gingerol exposure. Offspring weight also reduced by MEK exposure (*p* < 0.01) and rescued by gingerol. Similar outcomes were seen with markers of kidney function and liver function in both dams and offspring. Apoptotic index and fibrosis score (in liver and kidney) were increased by MEK (*p* < 0.01) and rescued by gingerol albeit to a lesser extent than other outcomes. Lipid peroxidation thiobarbituric acid reactive substances (TBARS) increased in pups in kidney, liver, and brain (*p* < 0.01) by MEK and rescued by gingerol at gestational days 7, 14, and 21. Oxidative stress parameters in pups are also shown, but difficult to interpret as presented.
**Studies investigating toxicity of compounds extracted from** *** Zingiberaceae*** ** family**							
Inegbenebor, 2009a	Rat (Sprague-Dawley)	4 / 1	1.Control 2. 0.5 mg alligator pepper 3. 1.0 mg alligator pepper 4. 1.5 mg alligator pepper 5. 2.0 mg alligator pepper *Administered as a bolus dose.	Not specified	Gestational weight gain	Birth weight	At even the lowest dose of alligator pepper, gestational weight gain was reduced by 50% (*p* < 0.01). Mean litter weight was reduced only in the 2.0 mg dose group (*p* < 0.001).
Inegbenebor, 2009b	Rat (Sprague-Dawley)	1 / 1	1. control 2. 50 mg alligator pepper / 20 g chow *Administered once on fourth day after mating	Once on fourth day after mating	Gestational weight gain Toxicity		Gestational weight gain was reduced by > 50% in experimental group (*p* < 0.001). Significant toxicity noted. No litters born to dams from experimental group.

### Characteristics of included studies

Of the 12 included studies (Table 1), a majority (75%) investigated multiple interventions including comparing different *Zingiberaceae* preparations and varying administration schedules including: *Zingiber officinale roscoe* (ginger/ginger extract/ginger aqueous extract), specific gingerol extract fractions (8.3%), *Zingiberaceae aframomum melegueta* (alligator pepper) (6.7%), and alpinetin (*Alpinia katsumadai hayata*). The studies were published between 2000 and 2022, with the majority (75%) being conducted in the last decade. Geographically, most of the studies (33%) were conducted in Saudi Arabia; followed by China (25%), the United States (17%), Egypt (8%), Nigeria (8%), and Japan (8%).

All studies featured rodent species such as rats (*Rattus norvegicus*, Wister, Sprague-Dawley) and mice (*Balb/c* and *ICR-CD1*). *Zingiberaceae* preparations were administered in different ways, including intraperitoneal injections, oral gavage, mixed into food, or included in drinking water. The preparation method for a majority of the studies included drying and crushing ginger rhizomes into a powder that was added to feed or dissolving ginger rhizomes with boiling or distilled water into an aqueous extract. Inegbenebor (2009a and 2009b) employed crushed alligator pepper seeds from an herbaceous plant species in the ginger family, that was then added to distilled water to make an aqueous extract which was administered via intra-peritoneal injection or mixed with rat feed [10, 11]. Othman (2022) treated mice with a gingerol-containing fraction of ginger rhizome extract containing [6]-gingerol (47.9%), [8]-gingerol (5.6%), [10]-gingerol (3.8%), and [8]-shogaol (1.2%) [12]. Weidner (2001) used “EV.EXT 33” described as “a patented standardized ethanol extract of dry rhizomes of *Zingiber officinale r*oscoe (*Zingiberaceae*)” also containing [6]-gingerol, [8]-gingerol, [10]-gingerol, and [8]-shogaol [13]. Weidner (2001) also used sesame oil as an oral administration vehicle for ginger to enhance bioavailability. Hajin (2013) used alpinetin purchased from the National Institute for Control of Pharmaceutical and Biological Products (Beijing, China). The dosages and treatment durations varied widely, starting from pre-pregnancy, early pregnancy (6 days of a 21-day rodent gestation) and continuing until late gestation or through lactation. In some cases, the ginger or ginger extract treatment was administered in addition to other toxic exposures such as pesticides, pharmaceutical agents, and heavy metals [13].

Maternal outcomes reported included gestational weight gain, the prevention of toxicity, defined as adverse physiological effects such as cellular fibrosis, apoptosis, and organ damage (e.g., liver, kidney) when *Zingiberaceae* preparations were administered alongside harmful substances (pesticides or heavy metals), and lactation outcomes defined as improved anti-inflammatory properties in mammary tissues and reduced tissue damage. Toxicity in these studies was mitigated by the protective effects of ginger, reducing cellular apoptosis and improved organ histology and function, as well as normalized growth markers in maternal and fetal tissues. Fetal outcomes reported included fetal death, growth metrics including birth weight and crown-rump length, and indicators of toxicity, such as histological damage to organs (liver, kidney, cardiac) or elevated apoptosis in fetal tissues.

### Risk of Bias assessment

**Fig. 2:**
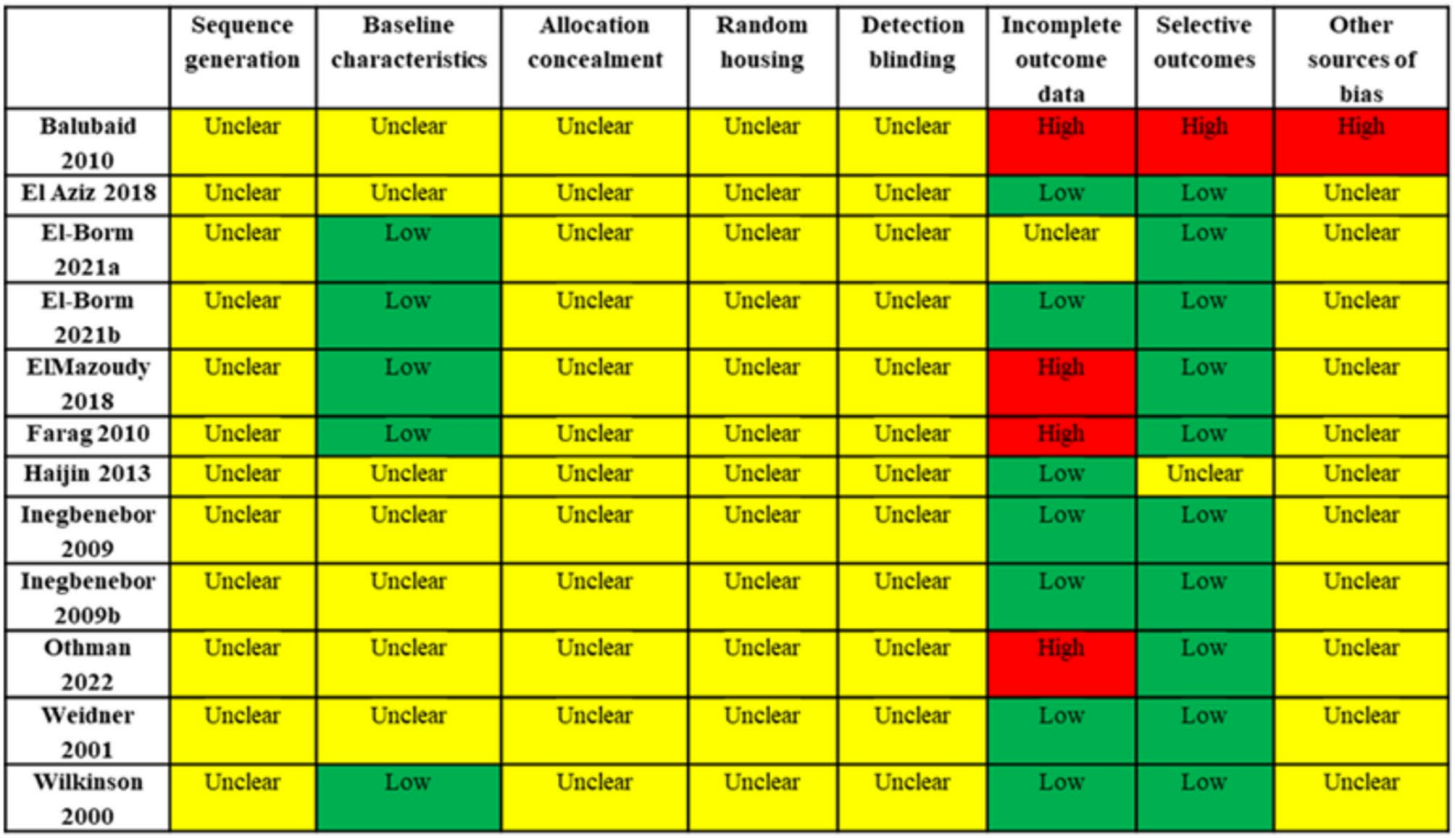
Risk of bias of selected articles. Risk of bias was performed as directed by the Systematic Review Center for Laboratory Animal Experimentation (SYRCLE) tool [6]

Risk of bias was assessed using the SYRCLE ROB tool (Fig. 2) [8]. None of the included studies included a description of methods (if any) used for randomization. Therefore, sequence generation was considered unclear for all studies. 42% of studies listed strain, age, and weight of animals [14–18] and were judged to be at low risk of bias for baseline characteristics. The remaining studies, which did not specify strain or did not specify age and weight of animals at baseline [2, 10–13, 19], were considered unclear risk of bias in baseline characteristics. None of the included studies described if there was animal coding (i.e. allocation concealment) or how it was performed; therefore, allocation concealment was considered unclear for all studies. Similarly, no studies described how animals were housed (placement, handling, etc.), resulting in unclear risk of bias for random housing. Housing conditions are expected to have the most significant effects on animal behavior outcomes and less likely to affect the physiological / biochemical outcomes as measured in the included studies (weight gain, implantations, fetal growth, apoptosis–primary outcomes of interest in of this analysis), therefore this was not considered an important limitation in included studies. No studies included a description of if outcome assessors were blinded to treatment assignment, leaving all studies at unclear risk of bias for detection blinding.

More than half (58%) of studies were considered low risk of bias for incomplete outcome data because the number of animals reported in outcome figures/tables was consistent with the number of animals described as randomized to each group and consistent across outcomes [10, 11, 13, 15, 18, 19]. One study was judged unclear risk of incomplete outcome data because presentation of histological data did not indicate how many animals the images represent, or whether they are from only selected animals [14]. 33% of studies were considered high risk of bias for this domain due to a lack of description of animals reported in presented data [2] or for discrepancies in animal numbers reported across outcomes without a description of why numbers differed between outcomes [12, 16, 17]. No studies reported study protocol registration, though this is not yet widely performed for animal trials or required by peer-reviewed journals. Therefore, 83% of studies were considered to be low risk of bias for selective outcomes if all experiments described in methods were also reported in results [10, 12–19]. One study was unclear for selective outcome bias [4] and one study was high risk of selective outcome reporting [2] for not adequately describing methods used (i.e. how morphological characteristics were assessed and other techniques), therefore not allowing for an assessment of whether experiments performed and reported outcomes matched. All studies except one [2] were judged unclear risk of “other” bias because they either (1) lacked description of animal diet (or used non-specified chow), or (2) included histological data shown only as representative images that are not quantified across the cohort. Balubaid (2010) was judged as high risk of other bias because animal diet was not mentioned, and most experimental methods were not described [2].

### Maternal outcomes

#### Gestational weight gain

More than half (66%) of identified studies examined the effects of *Zingiberaceae* preparations on gestational weight gain. Balubaid (2010) showed that ginger (*Zingiber officinale*) partially restored tetracycline-induced reduction in maternal body weight [2]. When ginger rhizome aqueous extract (250 mg) was co-administered with cadmium (Cd) (8.8 mg), the results were indicative of a partial protective effect rescuing Cd-induced decreases gestational weight gain [19]. Final mean gestational weight (± SD) for the Cd group (255.5 g ± 10.7) was lower than final mean gestational weight in the Cd with the ginger rhizome extract group (287.4 g ± 10.9, *p* < 0.0001) [19]. Gravid uterine and placental weight showed similar trends among the ginger rhizome extract group and Cd with ginger rhizome extract group. In another study, methyl ethyl ketone (MEK) exposure resulted in significant reduction in gestational body weight (± SD) (152 ± 2.3 g) compared to the MEK and gingerol-treated group (168 ± 6.8 g, *p* < 0.001), restoring total body weight to untreated control levels [12]. Weidner (2001) found ginger (Eurovita extract 33, EV.EXT 33) at the highest dose (1000 mg) statistically reduced total body weight (± SD) (317.3 g ± 20.6 compared to 301.3 g ± 22.9) at some time points during gestation (day 15 and 20), however final gestational weight gain was not different between EV.EXT 33-treated and control groups at the end of gestation, even at the highest doses. Additionally, there was no significant difference to reproductive outcomes (e.g., number of implantations, live fetuses, litter size) and fetal skeletal alterations as a result of EV.EXT 33 treatment [13]. Farag (2010) found that combinations of ginger with lead or pesticides increased maternal body weight gain in days 6–20 of pregnancy compared to pesticide- or lead (Pb)-treated animals, suggesting a mitigation in toxicity in the ginger-treated groups. Inegbenebor (2009a) found that the administration of alligator pepper reduced gestational weight gain. More specifically, alligator pepper administered via intra-peritoneal injections as an aqueous extract at low doses (0.5 mg, 1 mg, 1.5 mg, 2.0 mg and 20 mg) reduced gestational weight gain by > 50% (*p* < 0.01, *p* < 0.001) [10]. Among the five experimental groups there was a significant reduction in gestational weight gain (50–75 g) when compared to the control group (175–200 g, *p* < 0.05). Authors attributed the weight to loss of appetite of diuretic effect of alligator pepper. However, Inegbenebor (2009b) reported that experimental groups given 50 mg of alligator pepper with 20 mg of chow experienced a decline in weight gain after two weeks (52.6 ± 19.46 g, *p* < 0.0001) and blood-stained vaginal discharge which could be indicative of possible toxicity or pregnancy maintenance compared to the control group (137.5 g ± 17.66) [11].

### Toxicity prevention and protective effects

In addition to the effects of toxins ginger on maternal on weight gain, 33% of identified studies tested the effect of *Zingiberaceae* preparations to mitigate maternal and fetal gestational and/or developmental outcomes due to toxicities when ginger was co-administered with harmful substances. El-Borm (2021a) demonstrated that a water extract of ginger (200 mg/kg) could mitigate labetalol-induced cardiac toxicity in rats, specifically fetal cardiac tissues during organogenesis. Labetalol (300 mg/kg), an anti-hypertensive drug typically used during pregnancy, increased early apoptotic rates in maternal cardiac muscle and fetal cardiac cells to 16.71 compared to the control group (1.25%); additionally, there were increases in late apoptotic (22.73%) and necrotic cell rates (18.61%) in fetal cardiac cells. Co-administration of ginger with labetalol rescued labetalol-induced adverse histological and pathological ultrastructural and molecular effects to the heart and cardiac tissues to nearly control levels. In a similar study [15], labetalol increased apoptosis in maternal cardiac muscle (as measured by the pro-apoptotic protein marker caspase-3 immunohistochemistry and increased caspase-3 expression) and decreased placental weight [15]. The administration of ginger at a dose of 200 mg/kg one hour after labetalol ameliorated these adverse effects, leading to improved cardiomyocyte structure and recovery of myofibril architecture (based on quantification of six cardiac muscles of female rat in photomicrographs).

Othman 2022 reported that administration of a gingerol-containing extract fraction (25 mg/kg) exhibited a protective effect against MEK toxicity (350 mg/kg) specifically noting a significant reduction in cellular fibrosis and apoptosis of all organ tissues [12]. Weidner (2001) reported gingerol (EV.EXT 33) caused no differential effects on maternal reproductive outcomes including uterine and placental weight, number of early or late resorptions, or litter size at any dose (100, 333, and 1000 mg/kg) [13]. Wilkinson (2000) also reported maternal treatment with ginger tea caused no significant difference in gestational weight gain, uterine, liver, kidney, or placental weight, but ginger treatment did increase fetal reabsorptions (10%, *p* < 0.05) [18].

### Lactation outcomes

Haijin (2013) investigated the impact of alpinetin, a flavonoid found in ginger, on mammary gland physiology and immune responses. This study reported that alpenitin reduced lipopolysaccharide (LPS)-induced inflammatory cytokine levels and myeloperoxidase (MPO) activity in mammary tissue [6]. MPO activity, an indicator of neutrophil infiltration and consequent tissue damage, was reduced in the LPS and alpinetin (10, 25, and 50 mg/kg) groups compared to the LPS only group. Histopathological evaluations revealed alpinetin-treated mice exhibited less mammary gland damage and inflammatory cell infiltration compared to the LPS-induced mastitis models.

### Fetal and offspring outcomes

#### Mortality and growth metrics

Several (25%) identified studies reported impacts of *Zingiberaceae* preparations on fetal and offspring growth outcomes. Inegbenebor (2009a) reported that maternal alligator pepper intra-peritoneal treatment impacted average litter weight among the five experimental groups. There was a 20% decrease in mean litter weight in the group receiving the highest dose 2.0 mg dose compared to non-treated control (*p* = 0.001) [10]. In contrast, 50 mg of alligator pepper mixed with 20 g of chow administered to dams early in pregnancy (first trimester) resulted in total fetal mortality, with the treated dams producing no viable litters [11]. Wilkinson (2000) found that exposure to ginger influenced various fetal parameters. Specifically, male fetuses in the 50 g/L ginger tea exposure group showed a significant increase in crown-rump length (*p* < 0.01) and body weight (*p* < 0.01). Female fetuses also demonstrated a significant weight increase in the 20 g/L (*p* < 0.001) and the 50 g/L ginger tea exposure groups (*p* < 0.05), respectively. Furthermore, there was a significant increase in sternal ossification, a measure of bone formation, in fetuses exposed to ginger tea, especially at the lower dose of 20 g/L, suggesting enhanced skeletal development as a result of ginger tea exposure [18].

### Toxicity prevention and adverse effects of ginger

Several (33%) identified studies showed that *Zingiberaceae* preparations administered during pregnancy/lactation may modify adverse effects of toxic heavy metals and chemicals exposed in offspring. Othman (2022) found that gingerol (25 mg/kg) rescued MEK-induced reductions in offspring weight, hepatorenal function, cellular fibrosis, and apoptosis of in assessed organ tissues (kidney and liver) [12]. There was also significant improvement in cellular antioxidant enzyme reduced glutathione (GSH) and superoxide dismutase (SOD) enzyme concentrations, in fetal tissues. In the MEK-treated group, GSH levels, associated with apoptosis, decreased in the kidney across all time points (postnatal days 7, 14, and 21), particularly on day 14, dropping to approximately 20 nmol/gm (*p* < 0.001). The gingerol-treated group, however, showed a significant restoration of GSH levels to about 50 nmol/gm on day 14 (*p* < 0.001). A similar pattern was observed in the liver, where GSH levels in the MEK-treated group were lower on days 7 and 14 (30–40 nmol/gm) compared to controls (*p* < 0.001, *p* < 0.001), but the gingerol-treated group showed a significant increase by day 21 (*p* < 0.001). SOD activity in the kidney of the MEK-treated group was reduced on days 7 and 21 (20 U/gm and 30 U/gm, *p* < 0.01), while the gingerol-treated group demonstrated significant recovery across all time points, reaching nearly 80 U/gm by day 14 (*p* < 0.001). In the liver, MEK exposure led to substantial decreases in SOD activity on days 7 and 14 (30 U/gm and 20 U/gm, *p* < 0.01), whereas the gingerol-treated group showed recovery to approximately 60 U/gm by day 21 (*p* < 0.001). Peroxidase activity in the kidney was reduced in the MEK-treated group, particularly on days 7 and 21 (20 U/gm and 15 U/gm, *p* < 0.01 and *p* < 0.001). In contrast, the gingerol-treated group exhibited significant recovery across all time points, with levels returning close to control values, especially by day 14 (60 U/gm, *p* < 0.001). Similar trends were observed in the liver, with MEK exposure causing a reduction in peroxidase activity on days 7 and 14 (30 U/gm and 20 U/gm, *p* < 0.01), and the gingerol-treated group showing restored peroxidase activity, exceeding 50 U/gm by day 21 (*p* < 0.001). The study highlighted specific gingerol fractions including [6]-gingerol, [8]-gingerol, [10]-gingerol, and [6]-shogaol were associated with antioxidant, anti-fibrotic, and/or anti-apoptotic properties of ginger phenolics in combating MEK-induced teratogenicity (i.e. chemical-induced fetal growth impedance) [12].

El-Aziz (2018) reported that *Zingiber officinale* improved and/or prevented fetal liver and kidney histological changes indicating a protective effect against Cd exposure. More specifically, the authors noted nearly normal hepatocytes, formation of central vein hepatic cords, and less damaged kidneys compared to Cd exposure, though histological results were not quantified [19], limiting interpretation of these findings. Weidner (2001) reported gingerol (EV.EXT 33) did not cause skeletal malformations. However, while lower doses of Zingiber officinale roscoe extract have demonstrated protective effects against gestational weight gain and fetal development, El Mazoudy (2018) reported adverse impact of overall reproductive and fetal development at high doses (2,000 mg/kgbw). Specifically, *Zingiber officinale roscoe* decreased gestational weight gain, disrupted estrous cycle length and diestrus index, reduced food consumption at the highest ginger dose (2,000 mg), increased pre-post implantation losses, and decreased crown-rump length, all indicating potential adverse effects at high concentrations. Although teratogenesis was not observed, these findings indicate *Zingiber officinale roscoe* at high doses can disrupt implantation and development [16].

### Subgroups and sensitivity analyses

Qualitative subgroup and sensitivity analyses were not possible due to a lack of more than one study employing the same *Zingiberaceae* preparation in any given species. Narrative subgroup and sensitivity analyses were conducted to explore the variability in Zingiberaceae preparations effects based on species (stratified), dosage (linear; low, medium, high), and duration of supplementation (linear; short term vs. long term). Outcomes were influenced by the dosage and duration of *Zingiberaceae* preparations supplementation. Higher doses (50 mg alligator pepper or 2,000 mg/kg body weight *Zingiber officinale roscoe*) were associated with adverse outcomes including gestational weight loss (< 50%), maternal toxicity, fetal death, and developmental anomalies [9, 14]. In contrast, shorter durations and lower doses comparable to dietary or supplement exposure (< 200 mg/kg/day, roughly equivalent to common use human consumption of ginger supplements 1000 mg/kg) yielded positive effects without significant toxicity [14, 15]. Sensitivity analysis confirmed that lower dosages (< 200 mg/kg/day) were reported as consistently safer and beneficial across different outcomes [14, 15].

### Publication Bias

The potential for publication bias in this systematic review was considered through narrative synthesis of the included studies. Several smaller studies with non-significant or less favorable outcomes were identified, suggesting limited publication bias [10, 11, 13, 16, 18, 19].

## Discussion

The findings from this systematic review highlight several key outcomes regarding the effects of *Zingiberaceae* preparations during pregnancy and lactation in animal models, particularly in terms of gestational weight gain, fetal development, toxicity prevention, and reproductive outcomes. These outcomes suggest there are potential dose-dependent protective effects across the species studied including maintenance of gestational weight and mitigation of gestational and developmental toxicity (e.g., liver, kidney, heart function and structure, skeletal formation).

Studies demonstrated that rhizome-derived ginger preparations (*Zingiber officinale*) at moderate doses (< 200 mg/kg/day), counteracted gestational weight loss and developmental toxicities induced by harmful substances such as heavy metals or toxic chemicals [2, 12, 19]. Several studies focused on specific gingerol compounds ([6]-gingerol, [8]-gingerol, [10]-gingerol, and [6]-shogaol) with antioxidant, anti-fibrotic, and anti-apoptotic properties that were associated with combating cytotoxicity and teratogenic effects. Similarly, alpinetin has shown promising anti-inflammatory properties, particularly in the context of lactation [6].

Whole-ginger and ginger rhizome preparations may maximize efficacy of *Zingiberaceae*-derived compounds for pregnancy-related outcomes. For example, whole-ginger rhizome preparations appeared to target multiple pathways involved in oxidative stress, inflammation, and anti-apoptotic mechanisms to prevent of developmental toxicity [12–15]. However, the administered dose of whole-ginger and rhizome preparations is critical, as supplementation with alligator pepper was generally toxic [10, 11]. between Similarly, the mode of preparation for many extracts (ethanolic extract, water extract, etc.) appeared to mediate the potential benefits and/or harms of *Zingiberaceae* preparations.

Subgroup and sensitivity analyses revealed that dosage and duration of treatment may influence outcomes, offering a more nuanced understanding of the conditions under which *Zingiberaceae* preparations may be effective. The majority of the studies used oral supplementation (oral intragastric/gastric intubation, food/water intake) mimicking human dietary intake [12–15, 17, 18], only three studies used intra-peritoneal administration [6, 10, 11] which bypasses the digestive system and may improve bioavailability of the compounds, thereby complicating the application of their finding to human dietary contexts in which most nutritional interventions are administered orally.

Experimental animal model systems allow for more rapid data collection and a comprehensive understanding of complex interactions among maternal diet and maternal and offspring physiological outcomes. Identified studies suggested that maternal treatment with preparations from plants of the *Zingiberaceae* altered inflammatory signaling, apoptosis, and lipid peroxidation in maternal and offspring tissues. More targeted studies linking these favorable molecular outcomes to improvement in offspring development will deepen the mechanistic understanding of the effects of ginger on offspring physiology and the potential use as a protective agent during the perinatal time period.

### Strengths and limitations

This systematic review consolidated a varying range of studies providing a comprehensive overview of protective effects of *Zingiberaceae* preparations. The systematic approach and adherence to a priori registered protocols (e.g., PRISMA) contribute to strength of findings. Other strengths include employment of several databases for identification of studies and assessment of risk of bias using the SYRCLE tool. The heterogeneity and insufficient number of studies reporting comparable results proved to be a limitation for this review, as they prevented the performance of a robust meta-analysis and quantitative subgroup and sensitivity analyses. In addition, many studies exhibited high risk of bias due to inadequate reporting of methods (randomization, allocation concealment, lack of animal diet description), missing outcome data, or inconsistent data presentation (Fig. 2). This relatively high risk of bias across all studies limits our confidence in the overall strength of findings, generalizability, and potential for translation to the human context. Moreover, the relatively high risk of bias across all studies precluded interpretation of findings in which studies with high risk of bias were excluded.

Finally, the outcomes (reduction of nausea and vomiting) in human trials assessing the role for ginger or ginger extract in optimizing human maternal and offspring health [3] were not the same as found in the current review of animal model literature. This may reflect an inherent limitation in research involving rodents, which do not vomit [20]. The discrepancy between outcomes observed in animal trials (i.e. fetal toxicity, fetal development, gestational weight gain, etc.) and human trials (nausea and vomiting only) limits generalizability of animal models to the human context for pregnancy-related outcomes associated with use of ginger or extracts from plants of the *Zingiberaceae* family. Overall, the findings suggest that larger human trials may be the most effective way to advance current understanding of use of ginger preparations for pregnancy- and lactation-related outcomes including gestational weight gain, fetal/offspring development, and adverse outcomes.

## Conclusion

This systematic review highlights the significant protective effects of ginger or ginger extract of the *Zingiberaceae* family (i.e. phenolic compounds), and alpinetin against various gestational and developmental toxicities in animal models. The findings suggest that *Zingiberaceae* preparations, at appropriate doses, can mitigate adverse effects induced by toxic agents, thereby improving maternal and fetal outcomes in rodents. Translation of findings from animal model literature to the human context is complicated in this case by the disparate nature of the pregnancy-related outcomes comparing human and animal trials.

## Electronic supplementary material

Below is the link to the electronic supplementary material.

Supplementary Material 1

## Data Availability

All data generated during this study are included in this published article and its supplementary information files.

## Abbreviations

Cd: Cadmium
GSH: Glutathione
HPLC: High Pressure Liquid Chromatography
LPS: Lipopolysaccharide
MEK: Methyl Ethyl Ketone
MPO: Myeloperoxidase
OSF: Open Science Framework
PICO: Population Intervention Comparison and Outcome
PRISMA: Preferred Reporting Items for Systematic Reviews and Meta-Analyses
RoB: Risk of Bias
SOD: Superoxide Dismutase
SWiM: Synthesis Without Meta-analysis
SYRCLE: Systematic Review Center for Laboratory Animal Experimentation
