# Supplementary material for: Systematic review of the impact of ginger extract and alpinetin on pregnancy outcomes in animal models
Source: BMC Complement Med Ther. 2025 May 16;25:179. doi: 10.1186/s12906-025-04904-z (PMC12083149; doi:10.1186/s12906-025-04904-z)
Supplement: Supplementary file 1 — Supplementary Material 1 [file 12906_2025_4904_MOESM1_ESM.docx]

**Supplementary Material for “Systematic review of the impact of ginger extract and alpinetin on pregnancy outcomes in animal models”**

**Summary of Search Strategy Organized by Database:**

***Medline***

1, exp ginger/ or exp ginger extract/

2, (alpinetin or ginger).ti,ab,kw.

3, (Alpinia blepharocalyx or Alnus firma or Zingiberaceae).ti,ab,kw.

4, or/1-3

5, exp Pregnancy/ or exp Pregnancy Complications/ or exp Pregnancy Outcome/

6, (prenatal* or postnatal* or antenatal* or maternal or pregnan*).ti,ab.

7, exp Lactation/

8, (lactat* or breastfeed* or nursing).ti,ab.

9, exp Infant, Newborn/

10, (infant* or baby or babies or newborn* or neonate*).ti,ab.

11, or/5-10

12, (animals not humans).sh.

13, (mice or mouse or rat or rats).ti,ab.

14, in vitro study.ti,ab,kw.

15, preclinical.ti,ab,kw.

16, or/12-15

17, review.pt.

18, (medline or medlars or embase or pubmed or cochrane).tw,sh.

19, (scisearch or psychinfo or psycinfo).tw,sh.

20, (psychlit or psyclit).tw,sh.

21, cinahl.tw,sh.

22, ((hand adj2 search$) or (manual$ adj2 search$)).tw,sh.

23, (electronic database$ or bibliographic database$ or computeri?ed database$ or online database$).tw,sh.

24, (pooling or pooled or mantel haenszel).tw,sh.

25, (peto or dersimonian or der simonian or fixed effect).tw,sh.

26, (retraction of publication or retracted publication).pt.

27, or/18-26

28, 17 and 27

29, meta-analysis.pt.

30, meta-analysis.sh.

31, (meta-analys$ or meta analys$ or metaanalys$).tw,sh.

32, (systematic$ adj5 review$).tw,sh.

33, (systematic$ adj5 overview$).tw,sh.

34, (quantitativ$ adj5 review$).tw,sh.

35, (quantitativ$ adj5 overview$).tw,sh.

36, (quantitativ$ adj5 synthesis$).tw,sh.

37, (methodologic$ adj5 review$).tw,sh.

38, (methodologic$ adj5 overview$).tw,sh.

39, (integrative research review$ or research integration).tw.

40, or/29-39

41, 28 or 40

42, randomized controlled trial.pt.

43, (random$ or placebo$ or single blind$ or double blind$ or triple blind$).ti,ab.

44, (retraction of publication or retracted publication).pt.

45, or/42-44

46, ((comment or editorial or meta-analysis or practice-guideline or review or letter) not "randomized controlled trial").pt.

47, (random sampl$ or random digit$ or random effect$ or random survey or random regression).ti,ab. not "randomized controlled trial".pt.

48, 45 not (46 or 47)

49, exp cohort studies/

50, cohort$.tw.

51, controlled clinical trial.pt.

52, epidemiologic methods/

53, limit 52 to yr=1966-1989

54, or/49-51,53

55, exp case-control studies/

56, (case$ and control$).tw.

57, or/55-56

58, (case$ and series).tw.

59, case reports.pt.

60, (case$ adj2 report$).tw.

61, (case$ adj2 stud$).tw.

62, or/58-61

63, (ae or to or po or co).fs.

64, (safe or safety).ti,ab.

65, side effect$.ti,ab.

66, ((adverse or undesirable or harms$ or serious or toxic) adj3 (effect$ or reaction$ or event$ or outcome$)).ti,ab.

67, exp product surveillance, postmarketing/

68, exp adverse drug reaction reporting systems/

69, exp clinical trials, phase iv/

70, exp poisoning/

71, exp substance-related disorders/

72, exp drug toxicity/

73, exp abnormalities, drug induced/

74, exp drug monitoring/

75, exp drug hypersensitivity/

76, (toxicity or complication$ or noxious or tolerability).ti,ab.

77, exp Postoperative Complications/

78, exp Intraoperative Complications/

79, or/63-78

80, 4 and 11 and 16 and 79

***Embase (Ovid)***

1, exp ginger extract/ or exp ginger/

2, (alpinetin or ginger or Alpinia blepharocalyx or Alnus firma or Zingiberaceae).ti,ab,kw.

3, or/1-2

4, exp pregnant woman/ or exp pregnancy complication/ or exp pregnancy outcome/

5, (prenatal* or postnatal* or antenatal* or maternal or pregnan*).ti,ab.

6, exp lactation/

7, (lactat* or breastfeed* or nursing).ti,ab.

8, exp newborn/

9, (infant* or baby or babies or newborn* or neonate*).ti,ab.

10, or/4-9

11, exp animal experiment/

12, (animal$ not human$).sh,hw.

13, (mice or mouse or rat or rats).ti,ab.

14, in vitro study.ti,ab,kw.

15, preclinical.ti,ab,kw.

16, or/11-15

17, exp review/

18, (literature adj3 review$).ti,ab.

19, exp meta analysis/

20, exp "Systematic Review"/

21, or/17-20

22, (medline or medlars or embase or pubmed or cinahl or amed or psychlit or psyclit or psychinfo or psycinfo or scisearch or cochrane).ti,ab.

23, RETRACTED ARTICLE/

24, or/22-23

25, 21 and 24

26, (systematic$ adj2 (review$ or overview)).ti,ab.

27, (meta?anal$ or meta anal$ or meta-anal$ or metaanal$ or metanal$).ti,ab.

28, or/25-27

29, (random$ or placebo$ or single blind$ or double blind$ or triple blind$).ti,ab.

30, RETRACTED ARTICLE/

31, 29 or 30

32, (book or conference paper or editorial or letter or review).pt. not exp randomized controlled trial/

33, (random sampl$ or random digit$ or random effect$ or random survey or random regression).ti,ab. not exp randomized controlled trial/

34, 31 not (32 or 33)

35, exp cohort analysis/

36, exp longitudinal study/

37, exp prospective study/

38, exp follow up/

39, cohort$.tw.

40, or/35-39

41, exp case control study/

42, (case$ and control$).tw.

43, or/41-42

44, exp case study/

45, (case$ and series).tw.

46, case report/

47, (case$ adj2 report$).tw.

48, (case$ adj2 stud$).tw.

49, or/44-48

50, (ae or si or to or co).fs.

51, (safe or safety).ti,ab.

52, side effect$.ti,ab.

53, ((adverse or undesirable or harm$ or serious or toxic) adj3 (effect$ or reaction$ or event$ or outcome$)).ti,ab.

54, exp adverse drug reaction/

55, exp drug toxicity/

56, exp intoxication/

57, exp drug safety/

58, exp drug monitoring/

59, exp drug hypersensitivity/

60, exp postmarketing surveillance/

61, exp drug surveillance program/

62, exp phase iv clinical trial/

63, exp drug surveillance program/

64, exp phase iv clinical trial/

65, (toxicity or complication$ or noxious or tolerability).ti,ab.

66, exp postoperative complication/

67, exp Peroperative Complication/

68, or/50-67

69, 3 and 10 and 16 and 68

***Cab Abstracts***

1, (Alpinetin or ginger or Alpinia blepharocalyx or Alnus firma or Zingiberaceae).ti,ab.

2, (prenatal* or postnatal* or antenatal* or maternal or pregnan*).ti,ab.

3, (lactat* or breastfeed* or nursing).ti,ab.

4, (infant* or baby or babies or newborn* or neonate*).ti,ab.

5, 2 or 3 or 4

6, (mice or mouse or rat or rats).ti,ab.

7, in vitro study.ti,ab.

8, preclinical.ti,ab.

9, or/6-8

10, review.ti,ab.

11, (medline or medlars or embase or pubmed or cochrane).tw.

12, (scisearch or psychinfo or psycinfo).tw.

13, (psychlit or psyclit).tw.

14, cinahl.tw.

15, ((hand adj2 search$) or (manual$ adj2 search$)).tw.

16, (electronic database$ or bibliographic database$ or computeri?ed database$ or online database$).tw.

17, (pooling or pooled or mantel haenszel).tw.

18, (peto or dersimonian or der simonian or fixed effect).tw.

19, (retraction of publication or retracted publication).ti,ab.

20, or/11-19

21, 10 and 20

22, meta-analysis.ti,ab.

23, meta-analysis.ti,ab.

24, (meta-analys$ or meta analys$ or metaanalys$).tw.

25, (systematic$ adj5 review$).tw.

26, (systematic$ adj5 overview$).tw.

27, (quantitativ$ adj5 review$).tw.

28, (quantitativ$ adj5 overview$).tw.

29, (quantitativ$ adj5 synthesis$).tw.

30, (methodologic$ adj5 review$).tw.

31, (methodologic$ adj5 overview$).tw.

32, (integrative research review$ or research integration).tw.

33, or/22-32

34, 21 or 33

35, randomized controlled trial.ti,ab.

36, (random$ or placebo$ or single blind$ or double blind$ or triple blind$).ti,ab.

37, (retraction of publication or retracted publication).ti,ab.

38, or/35-37

39, cohort$.tw.

40, controlled clinical trial.ti,ab.

41, or/39-40

42, (case$ and control$).tw.

43, (case$ and series).tw.

44, case reports.ti,ab.

45, (case$ adj2 report$).tw.

46, (case$ adj2 stud$).tw.

47, or/43-46

48, (safe or safety).ti,ab.

49, side effect$.ti,ab.

50, ((adverse or undesirable or harms$ or serious or toxic) adj3 (effect$ or reaction$ or event$ or outcome$)).ti,ab.

51, (drug adj1 (monitor* or toxic* or hypersensit*)).ti,ab.

52, (toxicity or complication$ or noxious or tolerability or poison*).ti,ab.

53, ((Postoperative or Intraoperative) adj2 Complication*).ti,ab.

54, or/48-53

55, 1 and 5 and 9 and 54

***International Pharmaceutical Abstracts***

1, (Alpinetin or ginger or Alpinia blepharocalyx or Alnus firma or Zingiberaceae).ti,ab.

2, (prenatal* or postnatal* or antenatal* or maternal or pregnan*).ti,ab.

3, (lactat* or breastfeed* or nursing).ti,ab.

4, (infant* or baby or babies or newborn* or neonate*).ti,ab.

5, 2 or 3 or 4

6, (mice or mouse or rat or rats).ti,ab.

7, in vitro study.ti,ab.

8, preclinical.ti,ab.

9, or/6-8

10, review.ti,ab.

11, (medline or medlars or embase or pubmed or cochrane).tw.

12, (scisearch or psychinfo or psycinfo).tw.

13, (psychlit or psyclit).tw.

14, cinahl.tw.

15, ((hand adj2 search$) or (manual$ adj2 search$)).tw.

16, (electronic database$ or bibliographic database$ or computeri?ed database$ or online database$).tw.

17, (pooling or pooled or mantel haenszel).tw.

18, (peto or dersimonian or der simonian or fixed effect).tw.

19, (retraction of publication or retracted publication).ti,ab.

20, or/11-19

21, 10 and 20

22, meta-analysis.ti,ab.

23, meta-analysis.ti,ab.

24, (meta-analys$ or meta analys$ or metaanalys$).tw.

25, (systematic$ adj5 review$).tw.

26, (systematic$ adj5 overview$).tw.

27, (quantitativ$ adj5 review$).tw.

28, (quantitativ$ adj5 overview$).tw.

29, (quantitativ$ adj5 synthesis$).tw.

30, (methodologic$ adj5 review$).tw.

31, (methodologic$ adj5 overview$).tw.

32, (integrative research review$ or research integration).tw.

33, or/22-32

34, 21 or 33

35, randomized controlled trial.ti,ab.

36, (random$ or placebo$ or single blind$ or double blind$ or triple blind$).ti,ab.

37, (retraction of publication or retracted publication).ti,ab.

38, or/35-37

39, cohort$.tw.

40, controlled clinical trial.ti,ab.

41, or/39-40

42, (case$ and control$).tw.

43, (case$ and series).tw.

44, case reports.ti,ab.

45, (case$ adj2 report$).tw.

46, (case$ adj2 stud$).tw.

47, or/43-46

48, (safe or safety).ti,ab.

49, side effect$.ti,ab.

50, ((adverse or undesirable or harms$ or serious or toxic) adj3 (effect$ or reaction$ or event$ or outcome$)).ti,ab.

51, (drug adj1 (monitor* or toxic* or hypersensit*)).ti,ab.

52, (toxicity or complication$ or noxious or tolerability or poison*).ti,ab.

53, ((Postoperative or Intraoperative) adj2 Complication*).ti,ab.

54, or/48-53

55, 1 and 5 and 9 and 54

**Supplementary Table 1. Characteristics of studies excluded during full text screening.**

| **Author and year** | **Title** | **Reason for exclusion** |
| --- | --- | --- |
| Booth 2010 | Oral dose-ranging developmental toxicity study of an herbal supplement (NT) and gallic acid in rats | Wrong intervention (consisted of an herbal supplement blend) |
| Chrubasik 2005 | Zingiberis rhizoma: a comprehensive review on the ginger effect and efficacy profiles | Wrong study design (narrative review) |
| Hosseini 2015 | Effect of alcoholic extract of Ginger during fetal life and breastfeeding on serum level of testosterone, LH, FSH and spermatogenic cells line in male mature offspring rats | Wrong language (full text not available in English language) |
| Hosseini 2015 | Effects of the alcoholic extract of ginger on sex hormone serum levels and ovarian follicles during pregnancy and lactation in the adult female offspring of rats | Wrong language (full text not available in English language) |
| Lete 2016 | The Effectiveness of Ginger in the Prevention of Nausea and Vomiting during Pregnancy and Chemotherapy | Wrong patient population (human, not animal model) |
| XuJianYa 2013 | Different effects of Rhizoma Pinelliae processed by ginger or decocting on gestation and embryonic development of mice | Wrong intervention (not from Zingerberaceae family) |
| XuJianYa 2017 | Effects of raw pinellia ternate and Ganjiang Renshen Banxia pill on reproductive related toxicity of mice | Full text not available |
